# Index Catalogue S. G. O.

**Published:** 1887-10

**Authors:** 


Index Catalogue S. G. O.—We have received from surgeon Billings in charge of the Surgean General's Library, Vol. VIII (Legier-Medicine—(naval.) When completed this will be a valuable record, and hundreds of years from now it will serve to resurrect papers buried in that great mass of literature, and they shall live long after their authors have been forgotten. Dr. Billings is a benefactor, in this respect.

